# Telomeric *NAP1L4* and *OSBPL5* of the *KCNQ1* Cluster, and the *DECORIN* Gene Are Not Imprinted in Human Trophoblast Stem Cells

**DOI:** 10.1371/journal.pone.0011595

**Published:** 2010-07-14

**Authors:** Jennifer M. Frost, Ramya Udayashankar, Harry D. Moore, Gudrun E. Moore

**Affiliations:** 1 Clinical and Molecular Genetics Unit, Institute of Child Health, University College London, London, United Kingdom; 2 Institute of Reproductive and Developmental Biology, Imperial College London, London, United Kingdom; 3 Department of Molecular Biology and Biotechnology, University of Sheffield, Sheffield, United Kingdom; Ottawa Hospital Research Institute and University of Ottawa, Canada

## Abstract

**Background:**

Genomic imprinting of the largest known cluster, the *Kcnq1/KCNQ1* domain on mChr7/hChr11, displays significant differences between mouse and man. Of the fourteen transcripts in this cluster, imprinting of six is ubiquitous in mice and humans, however, imprinted expression of the other eight transcripts is only found in the mouse placenta. The human orthologues of the latter eight transcripts are biallelically expressed, at least from the first trimester onwards. However, as early development is less divergent between species, placental specific imprinting may be present in very early gestation in both mice and humans.

**Methodology/Principal Findings:**

Human embryonic stem (hES) cells can be differentiated to embryoid bodies and then to trophoblast stem (EB-TS) cells. Using EB-TS cells as a model of post-implantation invading cytotrophoblast, we analysed allelic expression of two telomeric transcripts whose imprinting is placental specific in the mouse, as well as the ncRNA *KCNQ1OT1*, whose imprinted expression is ubiquitous in early human and mouse development. *KCNQ1OT1* expression was monoallelic in all samples but *OSBPL5* and *NAP1L4* expression was biallelic in EB-TS cells, as well as undifferentiated hES cells and first trimester human fetal placenta. *DCN* on hChr12, another gene imprinted in the mouse placenta only, was also biallelically expressed in EB-TS cells. The germline maternal methylation imprint at the KvDMR was maintained in both undifferentiated hES cells and EB-TS cells.

**Conclusions/Significance:**

The question of placental specific imprinting in the human has not been answered fully. Using a model of human trophoblast very early in gestation we show a lack of imprinting of two telomeric genes in the *KCNQ1* region and of *DCN*, whose imprinted expression is placental specific in mice, providing further evidence to suggest that humans do not exhibit placental specific imprinting. The maintenance of both differential methylation of the KvDMR and monoallelic expression of *KCNQ1OT1* indicates that the region is appropriately regulated epigenetically *in vitro.* Human gestational load is less than in the mouse, resulting in reduced need for maternal resource competition, and therefore maybe also a lack of placental specific imprinting. If genomic imprinting exists to control fetal acquisition of maternal resources driven by the placenta, placenta-specific imprinting may be less important in the human than the mouse.

## Introduction

Genomic imprinting is the epigenetic phenomenon of parent-of-origin dependent monoallelic expression. It is conserved among placental mammals and comprehensive data exists on imprinting in the mouse and human (http://igc.otago.ac.nz; http://www.har.mrc.ac.uk/research/genomic_imprinting/maps.html). Appropriate expression of imprinted genes is vital during fetal development and their disruption results in congenital disorders, primarily of growth and neurological development [1]. The phenotypes displayed by mutant mouse models and human syndromes associated with a loss of imprinting of certain genes demonstrate, in many cases, conservation of imprinted gene function and regulation between mouse and man [2], [3]. The large imprinted *Kcnq1/KCNQ1* domain on mChr7/hChr11 is a notable exception to this.

At *Kcnq1/KCNQ1* in both mice and humans, maternal DNA methylation of the promoter (KvDMR) of a non-coding RNA, *Kcnq1ot1/KCNQ1OT1,* leads to its paternal-specific expression [4]. *Kcnq1ot1/KCNQ1OT1* transcripts recruit polycomb-group complexes, mediating repressive histone modification on the paternal allele, repressing the surrounding genes *in cis* [5]–[7]. Imprinting at this domain is tissue specific. On the maternal allele in the mouse embryo, the absence of repressive chromatin allows expression of the five genes immediately centromeric to *Kcnq1ot1*. In the mouse placenta, the repressive chromatin domain formed by *Kcnq1ot1* transcripts is larger than in the embryo, and encompasses an additional eight flanking genes, resulting in maternal specific expression of a total of 13 transcripts [8]. In humans, only imprinting of the five central genes is evolutionarily conserved, and the eight flanking genes are expressed biallelically in the placenta [9]. This arrangement is consistent with differential methylation of the human KvDMR, paternal *KCNQ1OT1* expression and enrichment of repressive histone marks on the paternal allele at the central genes only [9].

This lack of conservation is not limited to the *KCNQ1* region. *DECORIN (DCN;* hChr12/mChr10) is an isolated imprinted gene, not associated with other imprinted transcripts. In the mouse, *Dcn* is ubiquitously expressed in embryos but its maternal-specific expression is limited to the placenta [10]. In human first trimester and term placenta, *DCN* expression is biallelic [9].

Early placentation events are more similar between phylogenetically divergent species than late ones. Gene expression profiles of the mouse placenta between E8.5 and E10.5 are characterised by evolutionarily ancient genes, common between different species, including humans. Expression profiles then gradually transfer to newer genes during mid to late gestation. By E15, the expression profile of the mouse placenta is enriched for rodent specific gene expression, and the human for primate specific gene expression [11]. Similarities restricted to early human and mouse gestation could include the phenomenon of placental specific imprinting, and therefore require analysis of early placental development.

Human embryonic stem (hES) cells have recently emerged as a useful model for placental development, providing the potential to study both the emergence of trophoblast from a precursor population and subsequent differentiation of these cells [12]. Conflict between paternal and maternal resources during pregnancy is thought, at least in some cases, to drive imprinting of genes [13]. The invasive character of endovascular trophoblast, and the direct contact of villous cytotrophoblast with the maternal bloodstream places these cells at the forefront of this conflict. Human trophoblast stem cells can be derived from hES cells following embryoid body formation (EB-TS cells). EB-TS cells are capable of differentiating to both villous and extravillous cytotrophoblast, and then subsequently to syncytiotrophoblast and endovascular trophoblast respectively [14]. We set out to characterise imprinting of the *KCNQ1* domain, analysing allelic expression of central, ubiquitously imprinted genes, and the flanking genes that are only imprinted in the mouse placenta, in the early human placenta using EB-TS cells as an *in vitro* model of human trophoblast precursors. The imprinting of the central genes is referred to here as ‘ubiquitous’ as during development these genes are imprinted where they are expressed (except for *KCNQ1* in heart), including in the placenta. This is in contrast to imprinting of ‘placental specific’ genes, which may be expressed widely but are only imprinted in the placenta. We analyse allelic expression of *DCN*, which is also only imprinted in the mouse placenta, and measure methylation at the putative imprinting control region for the *KCNQ1* domain, the KvDMR.

## Results

### Methylation of the KvDMR

**Figure 1:**
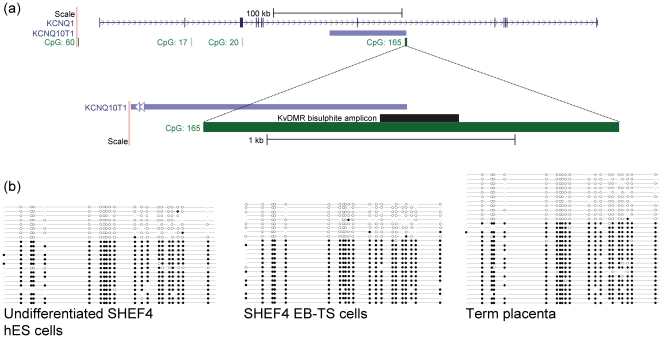
Analysis of DNA methylation at the KvDMR in hES cells, EB-TS cells and term placenta. (a) Layout of the KvDMR on human chromosome 11p15.5. The *KCNQ1OT1* promoter contains a CpG island, which also lies within intron 10 of *KCNQ1*. The CpG island is differentially methylated according to parental origin, with dense CpG methylation on the maternal allele. Paternal *KCNQ1OT1* expression results in silencing of surrounding genes, whereas maternal alleles remain active. (b) Methylation analysis of the KvDMR. Bisulphite PCR products containing 23 CpG dinucleotides were cloned and sequenced from human trophoblast stem cell DNA from H7 EB-TS cells, SHEF4 EB-TS cells, undifferentiated hES cells, and human term placenta tissue. CpG dinucleotides in the KvDMR are represented by open circles (unmethylated CpGs) and closed circles (methylated CpGs) on a string. Unique DNA strand clones for a single cloning experiment are shown for term placenta, undifferentiated hES cells and SHEF4 EB-TS cells, demonstrating the 35:65 ratio of unmethylated to methylated strands for each sample, strongly indicative of DMR maintenance. Each PCR and cloning step was repeated using a different bisulphite conversion. Overall, mean ratios of unmethylated to methylated strands were: Term placenta, 33:66; SHEF4 undifferentiated hES cells, 35:65; SHEF4 EB-TS, 41:59; H7 P8 EB-TS, 37:63; H7 P13 EB-TS, 35:65. The full set of data is provided in supplementary Dataset S1.

Methylation at the KvDMR, a CpG island in intron 10 of the *KCNQ1* gene, and the promoter for the *KCNQ1OT1* transcript, was analysed in hES and EB-TS cells and in term placenta (**Figure 1**). Bisulphite sequencing of SHEF4 hES cells, SHEF4 EB-TS cells at passage 15, H7 EB-TS cells both at passage 8 and at passage 13, and term placenta was carried out (see supplementary Dataset S1 for all clones). Figure 1 shows exemplar ‘lollipop’ diagrams generated by BiQ Analyser [15], showing methylated and unmethylated CpGs as closed and open circles respectively. There was a bias towards methylated stands, however, this was observed for each sample. In each sample a substantial proportion (on average 35%) of completely unmethylated DNA strands was observed, strongly indicative of maintenance of differential methylation at the KvDMR (**Figure 1**) This pattern of 35:65 unmethylated to methylated strands was seen in term placenta, undifferentiated hES cells and each EB-TS cell line. Strand specificity or parental origin could not be assigned due to a lack of informative SNPs in the EB-TS and hES cell lines and a lack of parental DNA.

### Allelic expression of imprinted genes in the *KCNQ1* cluster

The imprinted expression of transcripts in the human *KCNQ1* cluster was analysed in informative hES and EB-TS cell lines and first trimester placenta. A total of eight genetically distinct hES cell lines were analysed, and two EB-TS lines, one of which, SHEF4 EB-TS, was available alongside the hES cell line it was differentiated from, SHEF4 hES. The EB-TS cells had been cultured through several cycles of freeze-thawing for storage purposes, and through different passage numbers, so aliquots of cells were analysed after different freeze-thaw cycles and at different passages.

**Figure 2:**
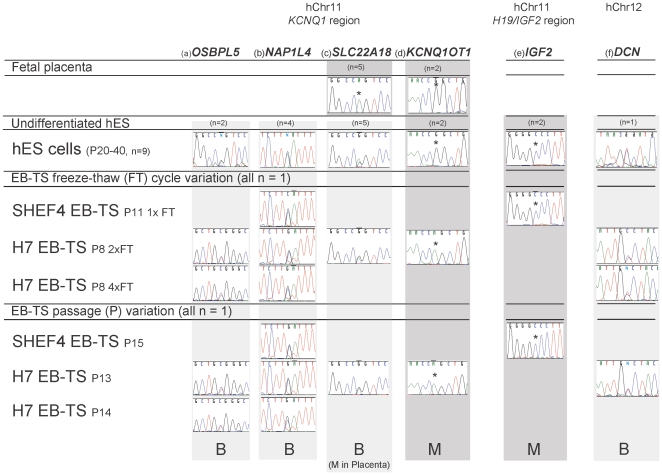
RT-PCR and sequencing of transcripts expressed in the KCNQ1 domain in informative EB-TS and hES cell samples. Allelic expression of genes in the *KCNQ1* domain, (a) *OSBPL5,* (b) *NAP1L4,* (c) *SLC22A18,* (d) *KCNQ1OT1,* plus (e) *IGF2* which is on the same chromosome arm but from a different imprinting domain, and (f) *DCN* on hChr 12, were analysed following RT-PCR and sequencing. Sequence chromatograms are displayed with mono- or biallelic expression represented as a single or double sequence trace at the polymorphic site, respectively. The polymorphic base is indicated by an asterix where monoallelic expression is shown. Telomeric genes, (a) *OSBPL5* and (b) *NAP1L4*, were biallelically expressed in all samples. (c) Central, ubiquitously expressed gene *SLC22A18* was monoallelically expressed in fetal placenta but biallelic in undifferentiated hES and EB-TS cells. Central gene (d) *KCNQ1OT1*, was monoallelic in EB-TS and hES cells. (e) *IGF2* was also monoallelically expressed in hES and EB-TS cells. (f) DCN on hChr 12 was biallelically expressed in EB-TS cells. B = Biallelic expression, M = monoallelic expression. Passage annotation: P8 indicates samples were analysed following eight passages from derivation. Samples which were freeze-thawed (FT) between passages were also analysed simultaneously and notated as follows: for example ‘P8 x1 FT’ meaning cells were analysed at passage 8, during which one freeze thaw cycle occurred. The number of EB-TS and undifferentiated hES cell lines found to be informative for each gene is shown (for example, n = 2). For the EB-TS cells, the number of genes which could be analysed was limited by informativity as only two genetically different lines were available. No differences were detected between the different samples. SNPs: *OSBPL5* rs935431, *NAP1L4* rs8505, *SLC22A18* rs1048046, *KCNQ1OT1* rs231357, *CD81* rs1049390, *IGF2* rs680 and *DCN* rs7441.

Of the flanking, placental specific genes, telomeric *NAP1L4 (rs8505)* and *OSBPL5 (rs935431)* were informative in EB-TS and hES cells. In each sample analysed, regardless of freeze thaw cycle or passage, both of these genes were expressed biallelically (**Figure 2a and b**). We therefore demonstrate a lack of imprinting, or even any allelic preference, of these transcripts in EB-TS cells, and show an identical expression profile in undifferentiated hES cells. These data show that the telomeric genes in the *KCNQ1* region are not imprinted in this *in vitro* model of early trophoblast development.

**Figure 3:**
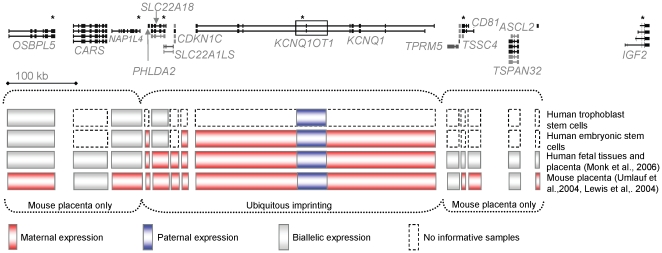
Genomic organisation of the imprinted gene cluster on hChr11. UCSC genome browser display of the KCNQ1 imprinted domain on human Chromosome 11p15.5 (human reference sequence NCBI Build 36.1). Genes are to scale (100 kb marker shown) oriented left to right from centromere to telomere and SNPs analysed are indicated by an asterix. The data presented here are summarised, where genes imprinted in the mouse placenta are not imprinted in human embryoid-body trophoblast stem cells but some central ubiquitously imprinted genes are monoallelically expressed, excepting *SLC22A18*. Genes maternally expressed, as demonstrated in previously published work, in mouse extraembryonic material and in human first trimester and term placenta and embryo are indicated in red, with the paternally expressed *Kcnq1ot1/KCNQ1OT1* in blue.

Of the central, ubiquitously imprinted transcripts, only *KCNQ1OT1* was informative in samples of hES and EB-TS cells. *KCNQ1OT1* was monoallelically expressed in hES, EB-TS cells and fetal placenta, as expected given the differential methylation at the KvDMR (**Figure 2d**). The other central genes were not informative in the available EB-TS cell lines, but were analysed in hES cells. *KCNQ1, PHLDA2* and *CDKN1C* were also monoallelically expressed in hES cells (data not shown). Given the monoallelic expression of *KCNQ1OT1*, it was intruiging to find that *SLC22A18*, a central ubiquitously imprinted gene that, whilst monoallelically expressed in first trimester placenta, was biallelically expressed in both hES and EB-TS cells (**Figure 2c**). Allelic expression of the *IGF2* gene, also encoded at 11p15.5 but under the control of a different transcriptional regulator than the KvDMR, was also analysed and found to be monoallelically expressed in EB-TS and hES cells (**Figure 2e**). These data and the genomic layout of this locus are illustrated in **Figure 3**.

### Allelic expression of *DCN*

*DCN*, encoded on hChr12 (mChr10) is widely expressed in the developing mouse, but maternal specific expression is limited to the placenta [10]. In human first trimester and term placenta, *DCN* expression is biallelic [9]. To extend the analysis of imprinting in EB-TS cells as a model for the early human placenta, the allelic expression of *DCN* was also analysed in these cells. *DCN* was expressed in undifferentiated hES cells and in EB-TS cells, and contained an informative exonic SNP for H7S14 EB-TS cells and one undifferentiated hES cell line. *DCN* expression was biallelic in undifferentiated hES cells and in H7S14 EB-TS samples at two different passage numbers, and in EB-TS cells following either two or four freeze/thaw cycles (**Figure 2f**).

## Discussion

Many more genes are imprinted in the mouse than in the human. This lack of conservation often occurs where there is placental specific imprinting [9], [16]. The growth and development of the placenta is central to the conflict theory over the different roles of parental nutrient provision. Vast differences between placentation, parity and the possibility of multiple paternity between humans and mice are likely to contribute to the differences between imprinting in the two species. As shown by genome-wide gene expression profiles, differences between mice and humans increase as prenatal development progresses [11].

To study the genes that are imprinted specifically in the mouse placenta in very early human placenta, a model was required that displayed a sufficiently early trophoblast phenotype. This analysis was carried out on trophoblast stem cells (EB-TS) which are representative of the earliest stages of placental development and capable of differentiation to both villous and extravillous trophoblast lineages [14]. Our results show that in these human trophoblast stem cells, *OSBPL5*, *NAP1L4* and *DCN* are biallelically expressed. These three genes are imprinted in the mouse placenta.

Until recently, the models available to study human placental development have all had significant drawbacks. Cells may be isolated from term placenta, or earlier, following termination. Most examples are already committed to villous trophoblast and only samples from very early gestation placenta are capable of differentiation to the extravillous lineage. Such primary trophoblast cultures have a finite lifespan in culture without viral transformation. Choriocarcinoma cell lines also maintain a villous trophoblast phenotype, and are capable of extensive proliferation, although their behaviour will inevitably display characteristics of malignancy [17]. The development of a system to derive trophoblast stem cells from human embryonic stem cells provides an *in vitro* model with invasive properties which are highly proliferative and readily available. In addition, EB-TS cells are capable of differentiation to both villous and extravillous trophoblast lineages, but without the genotype and phenotype changes associated with transformation, making these cells the best model for human early placental development.

We did identify small differences between imprinted gene expression in the placenta *in vivo* and in EB-TS cells, although differential methylation at the KvDMR seemed to be maintained in all samples, regardless of whether they were *in vivo* or *in vitro*. Whilst the ubiquitously imprinted gene *KCNQ1OT1* remained imprinted in both undifferentiated hES and EB-TS cells, as in fetal placenta, *SLC22A18* was expressed biallelically in all samples analysed. *SLC22A18* is maternally expressed in the human placenta throughout gestation, so although it has not been analysed *in vivo* during pre-implantation development, these data were surprising. As imprinted *SLC22A18* expression manifests only in peri-implantation stages in the mouse, it is possible that the differentiation state of EB-TS cells is too early for imprinted *SLC22A18* expression. Considerable evidence now exists for the biallelic expression of certain imprinted genes in undifferentiated human ES cell lines, however. Whilst for several genes this is polymorphic, *SLC22A18* is consistently reported to be biallelic in all hES cell lines [18], [19]. This indicates that EB-TS cells may harbour some selected features of the epigenotype of hES cells.

The influence of *in vitro* manipulation and culture limits the value of cell lines as a model for studies of imprinting. For studies in humans, however, these limitations are inescapable. As far as can be currently be investigated, and complimenting previous studies showing a lack of conservation between mouse and man [9] here we provide further evidence to suggest that humans do not have placenta-specific imprinting.

## Materials and Methods

### Sample collection and cell culture

#### Fetal placenta

Fetal placenta was collected from consenting women undergoing first-trimester termination of pregnancy, and forms part of the Moore Fetal Tissue bank. Collection was approved by the Hammersmith, Queen Charlotte's & Chelsea and Acton Hospitals Research Ethics Committee.

#### Human embryonic stem (hES) cells

Undifferentiated hES cell lines from Sheffield Univeristy were cultured in T25 flasks with 5 ml ES media containing 80% Knockout™ Dulbecco's modified Eagle's medium (Gibco), 20% Knockout™ Serum Replacement (Gibco), 1 mM L-glutamine, 0.1 mM β-mercaptoethenol (Sigma), 1% non-essential amino acids, supplemented with 20 ng/ml FGF-4. Cells were grown on a sub-confluent layer of Swiss-strain mouse embryonic fibroblasts (MEFs), except for hES cell line SHEF7, grown on human gonadal interstitial cell feeders. Colonies were dissociated for passaging using trypsin with 3 mm glass beads.

#### Human Trophoblast stem (EB-TS) cells

hES colonies were disrupted using 2 ml collagenase incubated at 37°C for 6 minutes, and removed from the flask surface using glass beads. The cell mix was added to a non-adhering bacterial plate in embryoid body (EB) media containing 80% Knockout DMEM, 20% knockout serum replacement, 1 mM glutamine, 0.1 mM β-mercaptoethenol and 1% non-essential amino acids. Cells forming EBs were cultured in EB medium for five days, before positive selection based on secretion of human chorionic gonadotrophin (hCG) and transferral to adherent plates containing MEFs. Cells were then cultured in trophoblast media consisting of RPMI (Sigma) supplemented with 20% fetal bovine serum (FBS), 1 mM Sodium Pyruvate, 100 µM β-mercaptoethenol, 2 mM LGlutamine and 50 µg/ml Pen/Strep. The cytotrophoblast stem cell lines received from Sheffield were derived from the SHEF4 line, and from the H7S14 sub-line, obtained from an H7 line gifted to the laboratory of Professor Peter Andrews by Dr J. Thomson.

### Analysis of DMR methylation

#### Bisulphite Sequencing

DNA from each sample was treated with sodium bisulphite and purified using the EZ DNA Methylation-Gold Kit™ (Zymo, CA), before amplification using primers specific for the KvDMR (primer sequences available on request). Two PCRs from different bisulphite conversions were carried out for each sample. DNA was tested for bisulphite conversion by PCR with the bisulphite-specific Prader Willi/Angelman Syndrome Imprinting Centre DMR primers in each case. Hotstar Taq polymerase (Qiagen, West Sussex, UK) was used for 45 PCR cycles. The three annotated SNPs within the KvDMR amplicon (rs3782064, rs7940500 and rs379976) were genotyped but neither were informative in the cell lines or the two placenta samples analysed.

Amplicons were ligated into the PGEM-T® Easy Vector (Promega) as per the manufacturers instructions. JM109 competent cells, of 10^8^ cfu/µg efficiency (Promega) were transformed and blue/white selected colonies amplified using M13 primers. Approximately 40 correctly-transformed colony PCRs per bisulphite PCR were sequenced (Applied Biosystems, CA), allowing calculation of bisulphite conversion rate and methylation profile of each CpG in the amplified region. Only strands with a C to T conversion efficiency over 90% were included in the analysis. BiQ Analyser software was used to generate ‘lollipop’ diagrams to illustrate methylated and unmethylated CpG sites along the amplified DNA strands [15]. All sequences included in the analyses differed in at least one aligned genomic ‘C’ position.

### Imprinting analysis

#### Genotyping and RT PCR

Genomic DNA extraction from snap frozen cell pellets was performed using standard phenol/chloroform extraction. Exonic single nucleotide polymorphisms (SNPs) were chosen based on their validation from the UCSC Genome Browser dbSNP build 129 (http://genome.ucsc.edu/cgi-bin/hgGateway) and expressed genes genotyped with respect to each SNP, primers and conditions as in Monk et al., 2006. Genotyping was performed on the following genes: *OSBPL5 (rs935431 and* rs2289998*), NAP1L4 (rs8505), PHLDA2* (rs13390), *CDKN1C (PAPA repeat), SLC22A18* (rs1048046, rs1048047), *KCNQ1* (rs1057128), *KCNQ1OT1 (rs231357, rs231359), TSSC4 (rs2522009), CD81 (rs10645), PHEMX (rs2074022)*, *ASCL2/MASH2* (rs2072072) - all *KCNQ1* cluster, hChr11. *IGF2* (rs680) - H19/IGF2 cluster, hChr11. *SLC22A2 (rs3127594, rs3219198, rs4646240) IGF2R* cluster, hChr6 and *DCN* (rs7441) hChr12. Total RNA from each cell line was extracted and reverse transcriptase PCR (RT-PCR) carried out as follows: Briefly, following TURBO DNase™ (Ambion) treatment, 1 µg total RNA was treated with DNaseI (Promega), primed with random hexamers and reverse-transcribed with Murine-Maloney Leukaemia virus (MMLV) reverse transcriptase (RT) (Promega). Templates created omitting the RT enzyme were included in the RT-PCR in each case. Where possible, given the requirement for amplicons to include validated exonic SNPs (db SNP Build 130), primers had been designed to cross intron-exon boundaries (9). RT-PCR was carried out on individual cDNA samples from each cell line. Genotypes and allelic expression were determined using a combination of Sanger sequencing (Applied Biosystems, CA) and restriction fragment length polymorphism (RFLP) analysis.

## Supporting Information

Dataset S1 Lollipop diagrams for each unique clone from the bisulphite sequencing of the KvDMR in hES, EB-TS and human placenta tissues.
(1.85 MB PPT)
