# Supplementary material for: Telomeric NAP1L4 and OSBPL5 of the KCNQ1 Cluster, and the DECORIN Gene Are Not Imprinted in Human Trophoblast Stem Cells
Source: PLoS One. 2010 Jul 14;5(7):e11595. doi: 10.1371/journal.pone.0011595 (PMC2904374; doi:10.1371/journal.pone.0011595)
Supplement: Dataset S1 — Lollipop diagrams for each unique clone from the bisulphite sequencing of the KvDMR in hES, EB-TS and human placenta tissues. (1.85 MB PPT) [file pone.0011595.s001.ppt]

## Slide 1
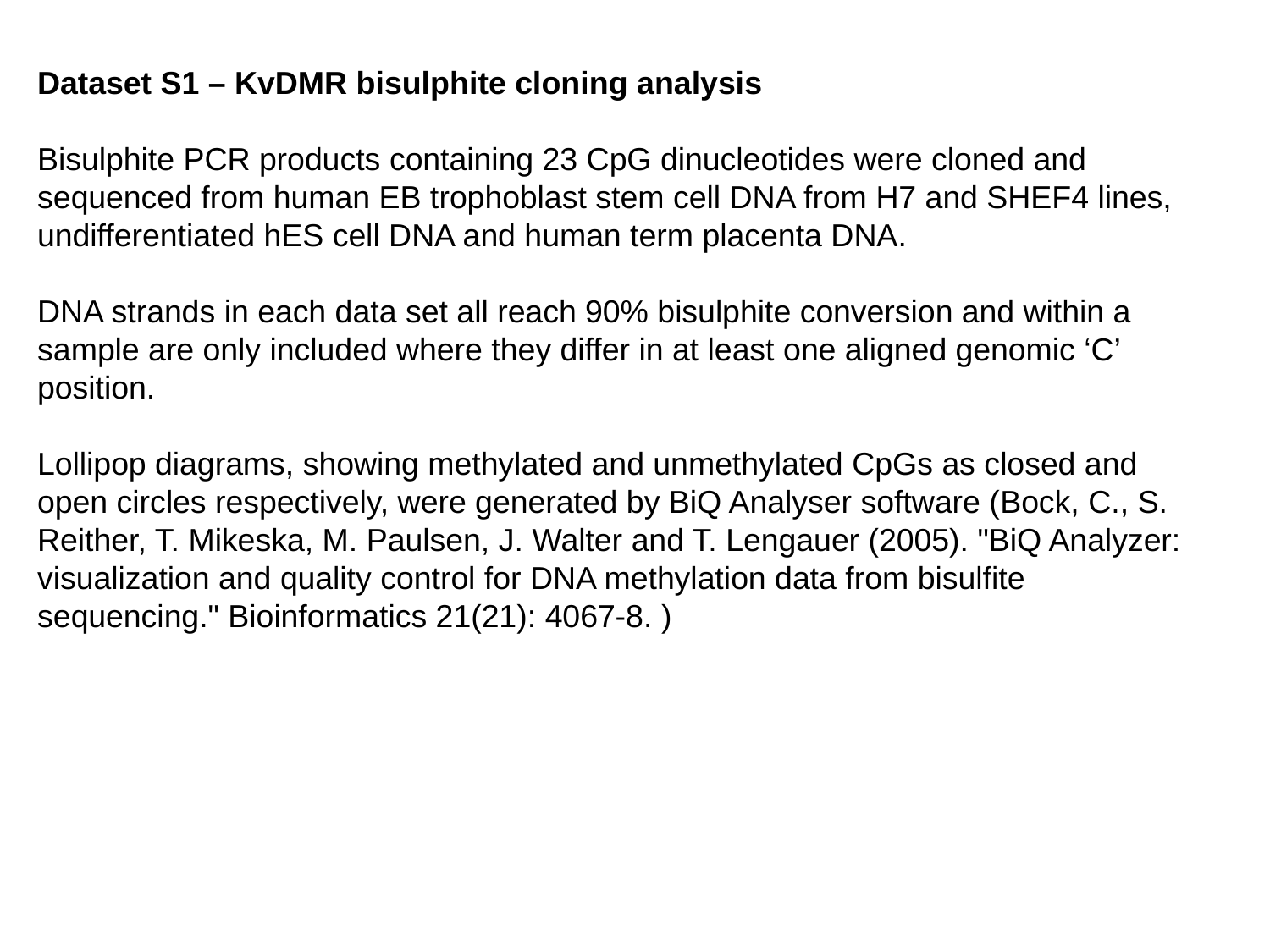

Dataset S1 – KvDMR bisulphite cloning analysis
Bisulphite PCR products containing 23 CpG dinucleotides were cloned and sequenced from human EB trophoblast stem cell DNA from H7 and SHEF4 lines, undifferentiated hES cell DNA and human term placenta DNA.
DNA strands in each data set all reach 90% bisulphite conversion and within a sample are only included where they differ in at least one aligned genomic ‘C’ position.
Lollipop diagrams, showing methylated and unmethylated CpGs as closed and open circles respectively, were generated by BiQ Analyser software (Bock, C., S. Reither, T. Mikeska, M. Paulsen, J. Walter and T. Lengauer (2005). "BiQ Analyzer: visualization and quality control for DNA methylation data from bisulfite sequencing." Bioinformatics 21(21): 4067-8. )

## Slide 2
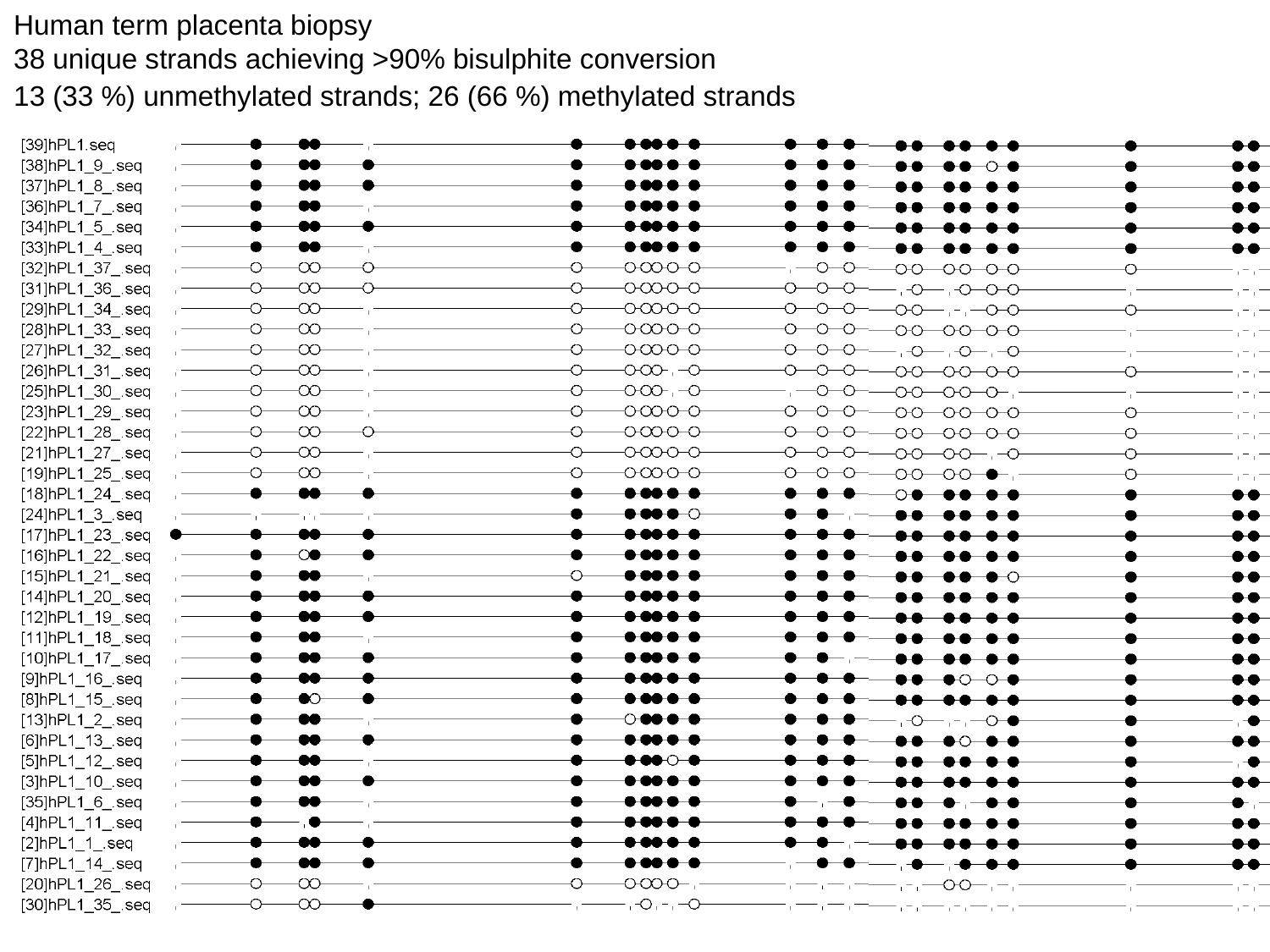

Human term placenta biopsy
38 unique strands achieving >90% bisulphite conversion
13 (33 %) unmethylated strands; 26 (66 %) methylated strands

## Slide 3
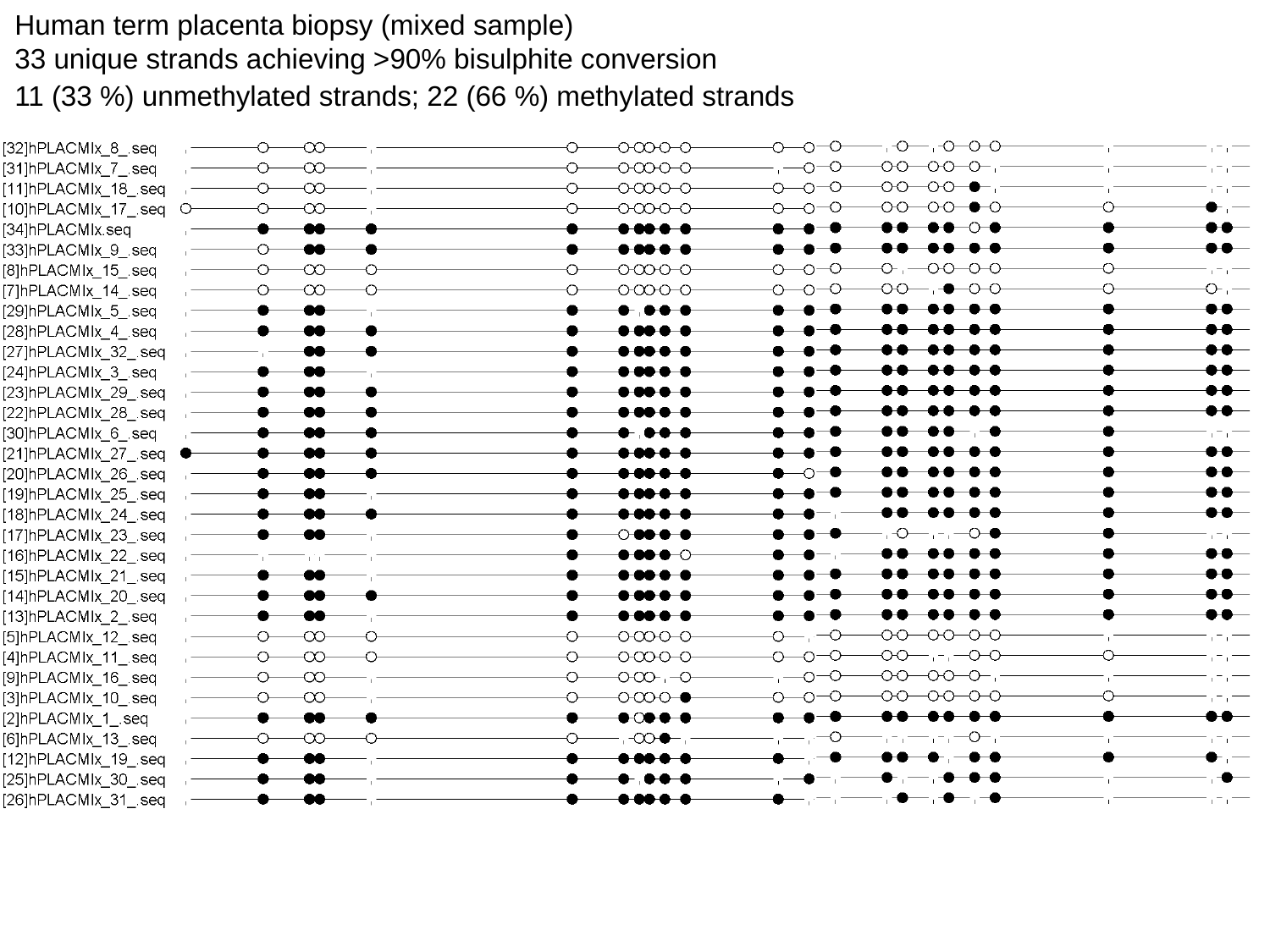

Human term placenta biopsy (mixed sample)
33 unique strands achieving >90% bisulphite conversion
11 (33 %) unmethylated strands; 22 (66 %) methylated strands

## Slide 4
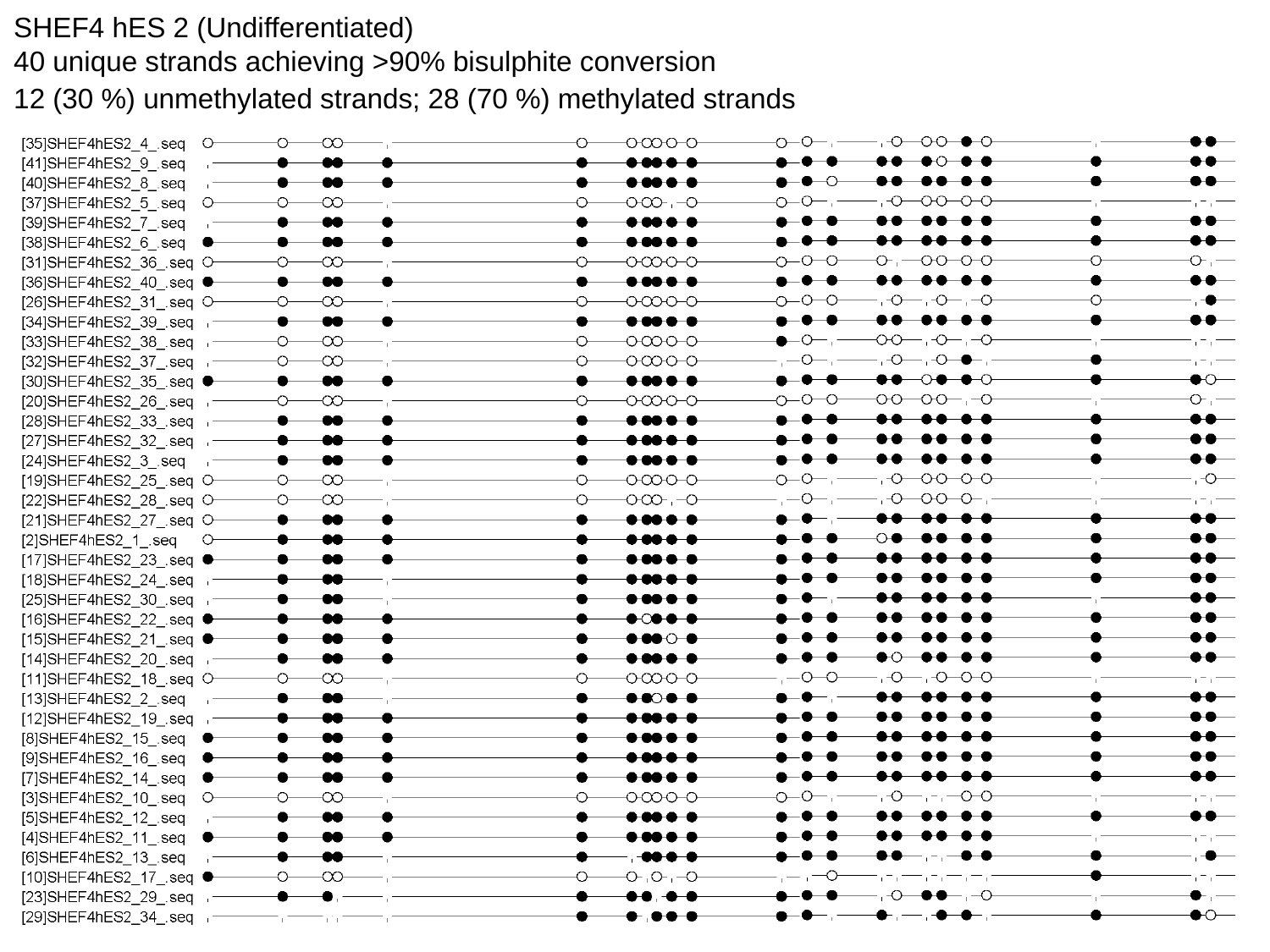

SHEF4 hES 2 (Undifferentiated)
40 unique strands achieving >90% bisulphite conversion
12 (30 %) unmethylated strands; 28 (70 %) methylated strands

## Slide 5
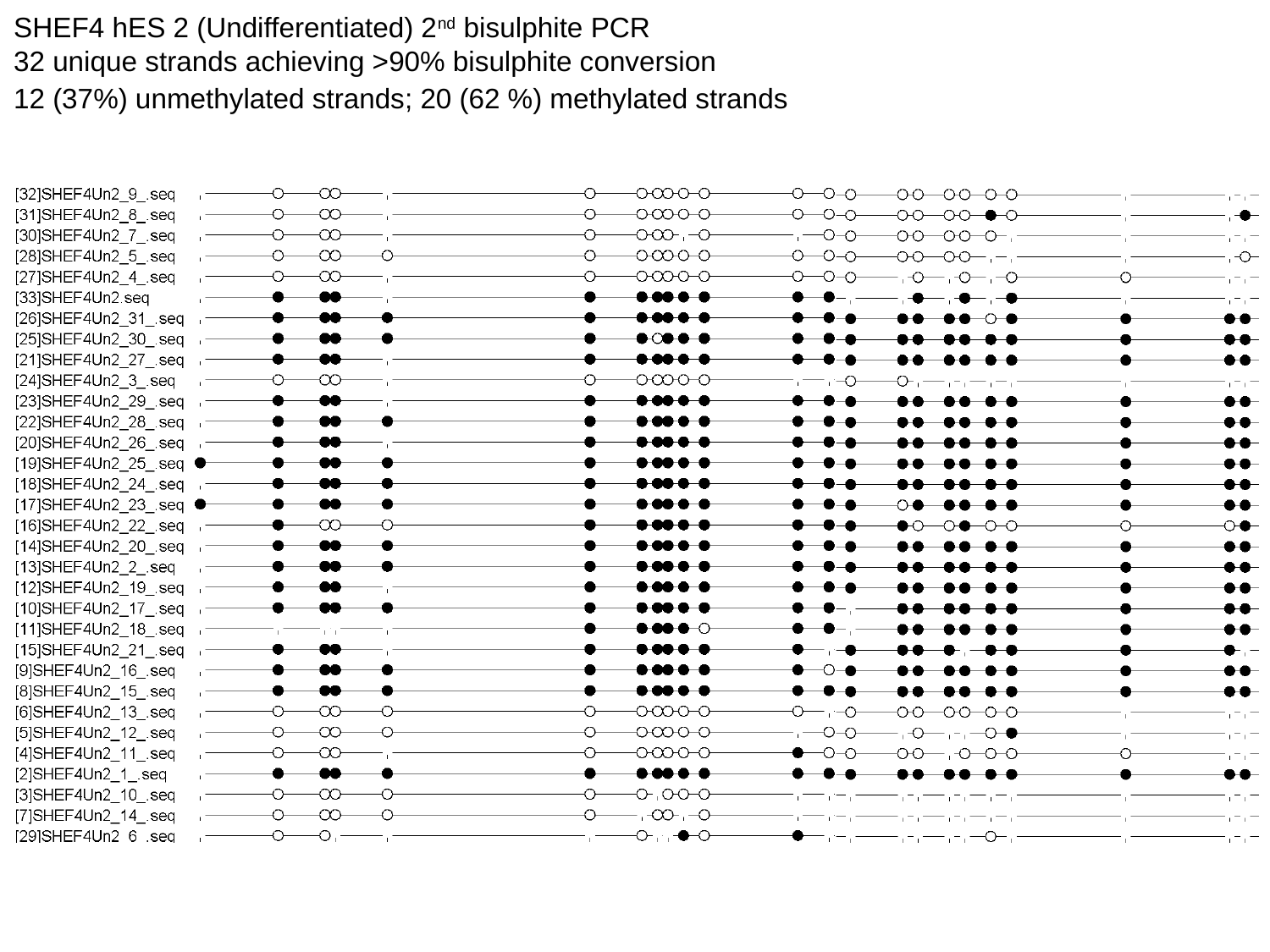

SHEF4 hES 2 (Undifferentiated) 2nd bisulphite PCR
32 unique strands achieving >90% bisulphite conversion
12 (37%) unmethylated strands; 20 (62 %) methylated strands

## Slide 6
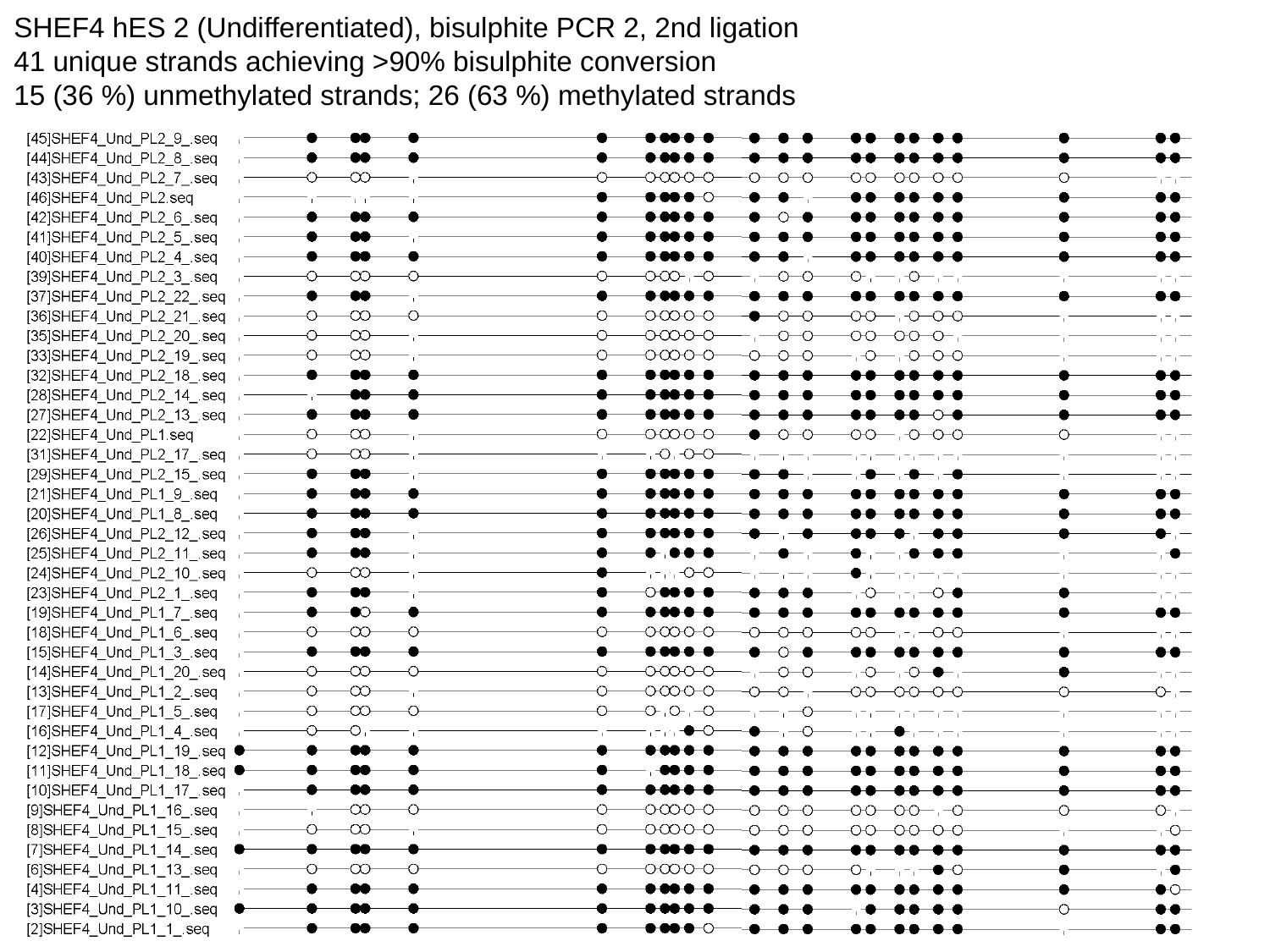

SHEF4 hES 2 (Undifferentiated), bisulphite PCR 2, 2nd ligation
41 unique strands achieving >90% bisulphite conversion
15 (36 %) unmethylated strands; 26 (63 %) methylated strands

## Slide 7
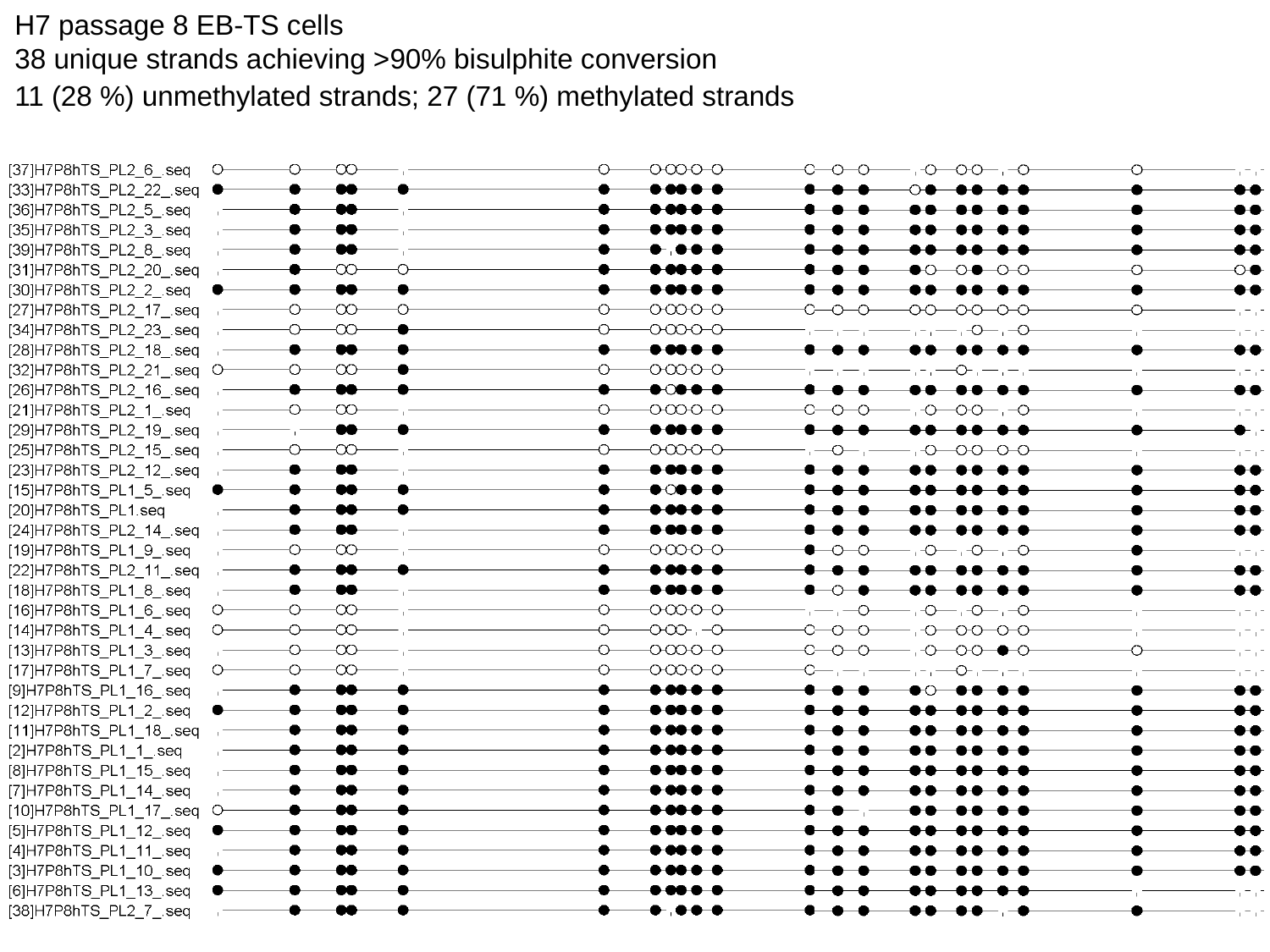

H7 passage 8 EB-TS cells
38 unique strands achieving >90% bisulphite conversion
11 (28 %) unmethylated strands; 27 (71 %) methylated strands

## Slide 8
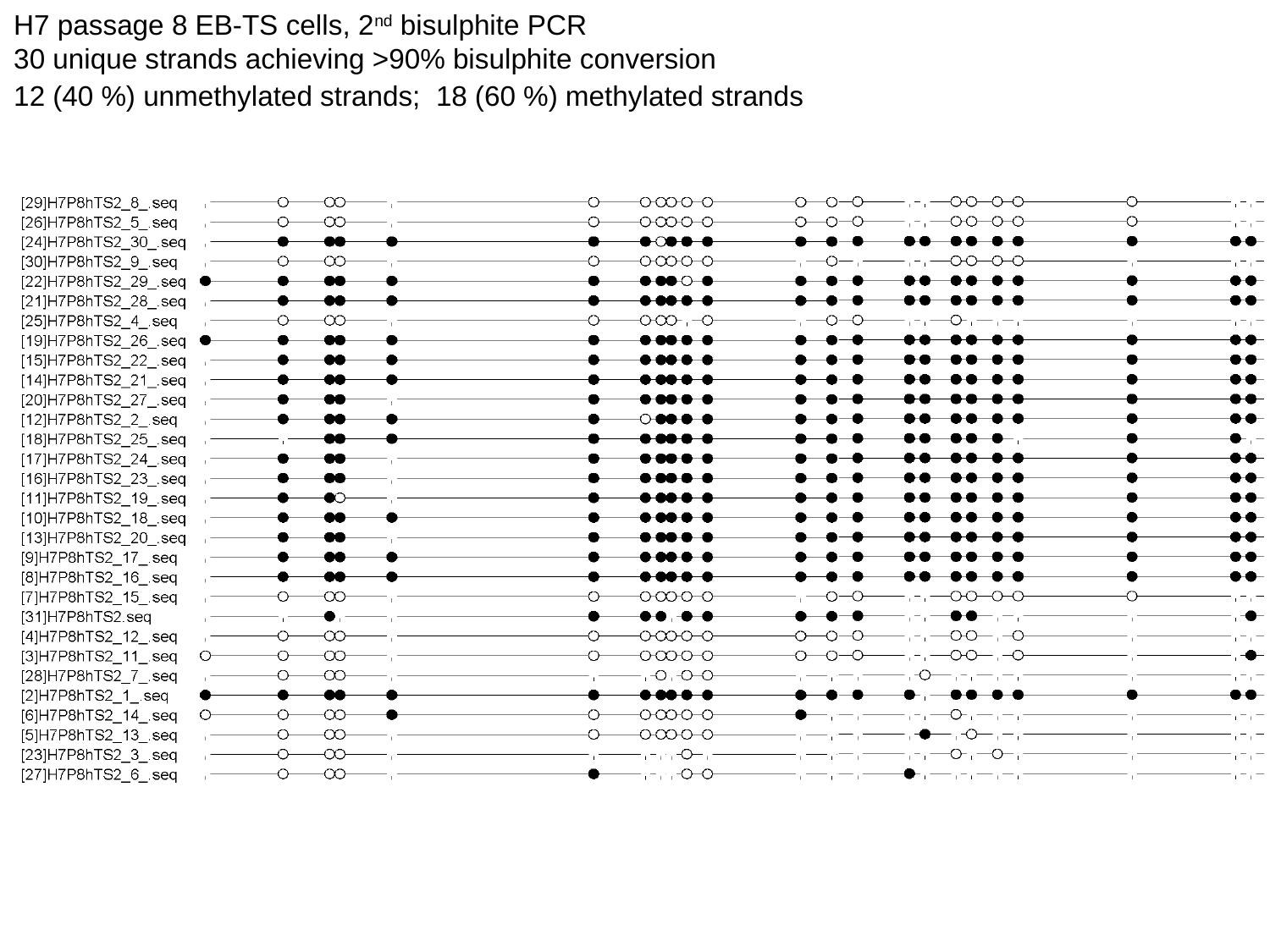

H7 passage 8 EB-TS cells, 2nd bisulphite PCR
30 unique strands achieving >90% bisulphite conversion
12 (40 %) unmethylated strands; 18 (60 %) methylated strands

## Slide 9
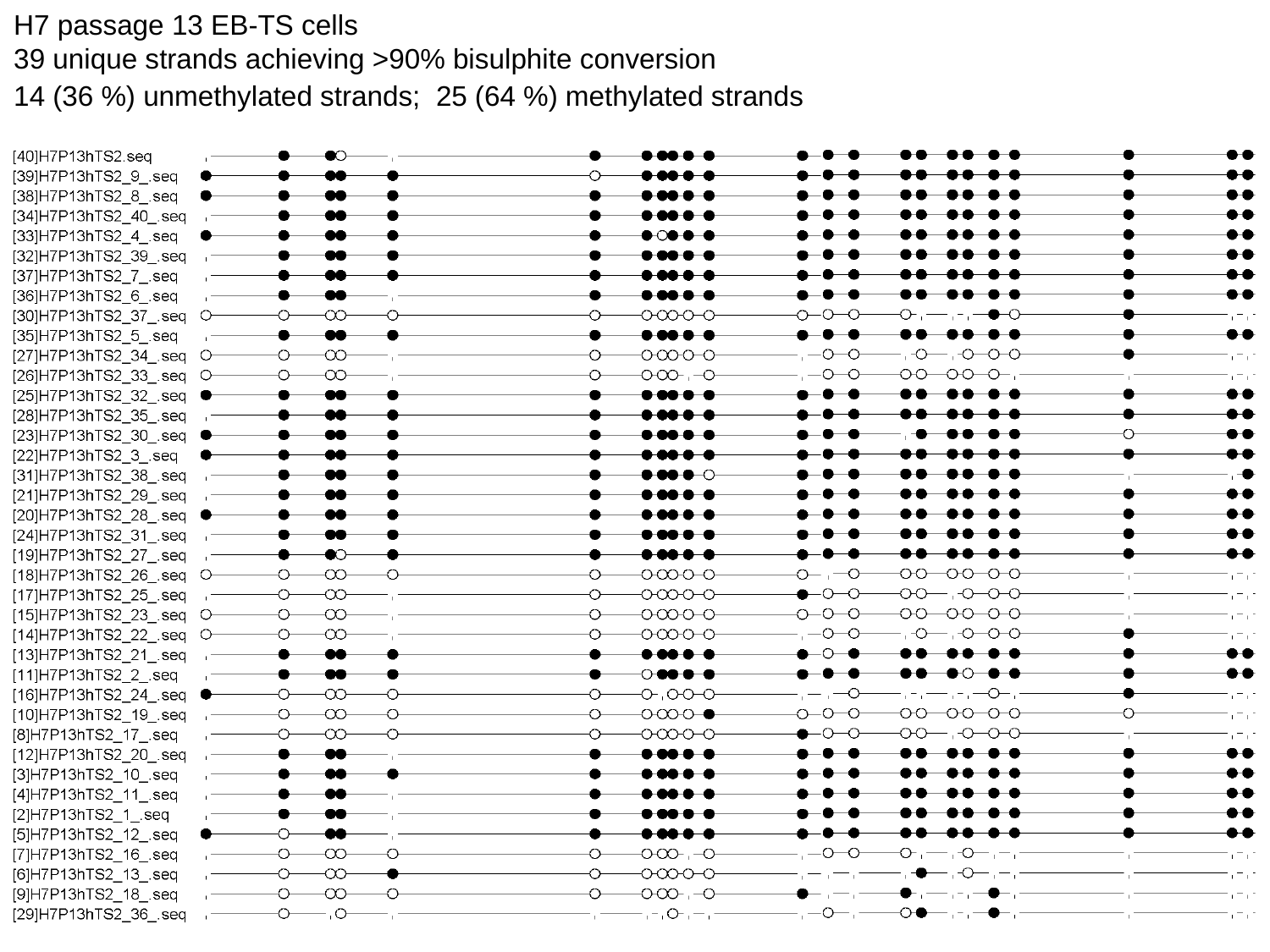

H7 passage 13 EB-TS cells
39 unique strands achieving >90% bisulphite conversion
14 (36 %) unmethylated strands; 25 (64 %) methylated strands

## Slide 10
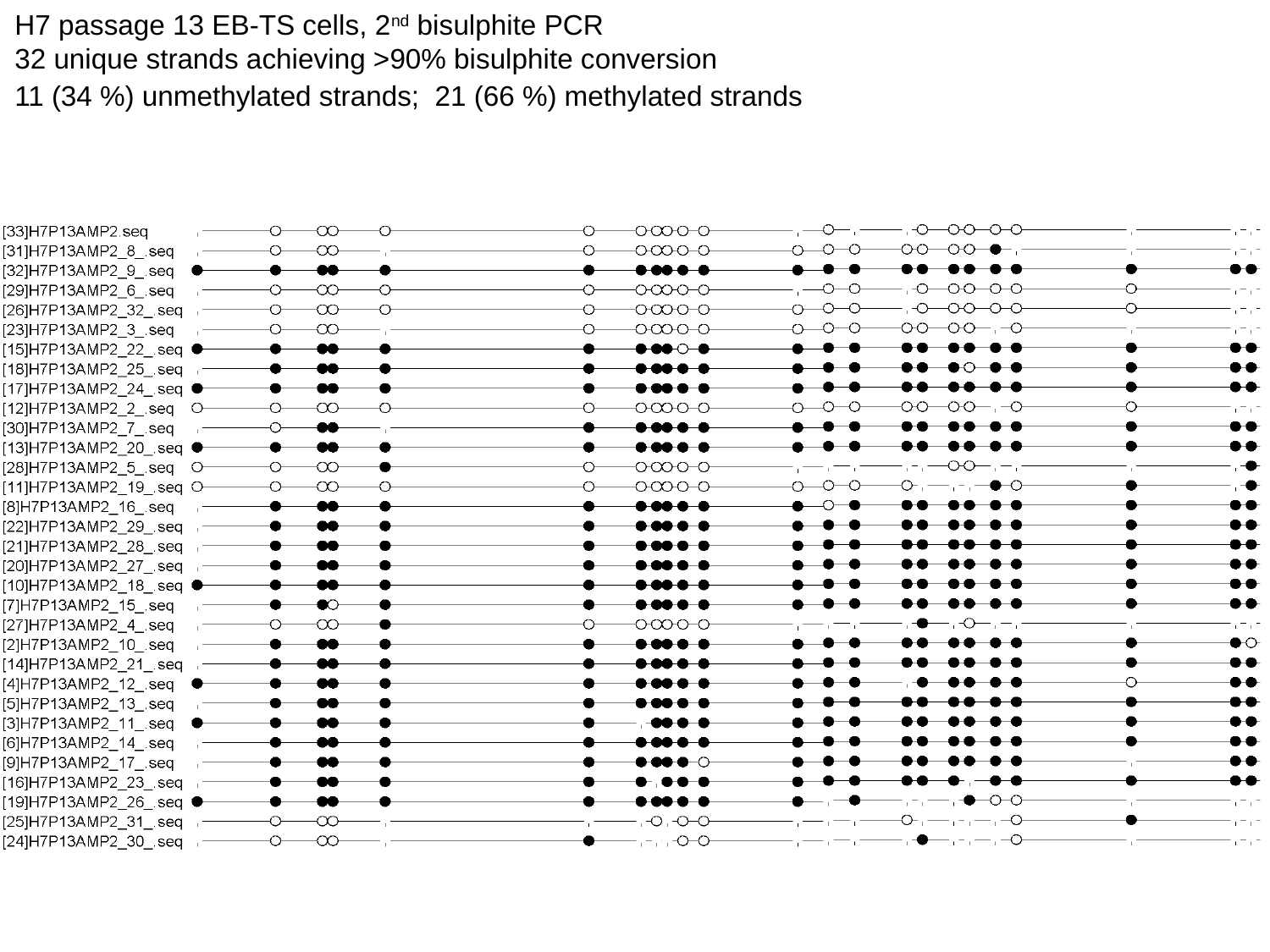

H7 passage 13 EB-TS cells, 2nd bisulphite PCR
32 unique strands achieving >90% bisulphite conversion
11 (34 %) unmethylated strands; 21 (66 %) methylated strands

## Slide 11
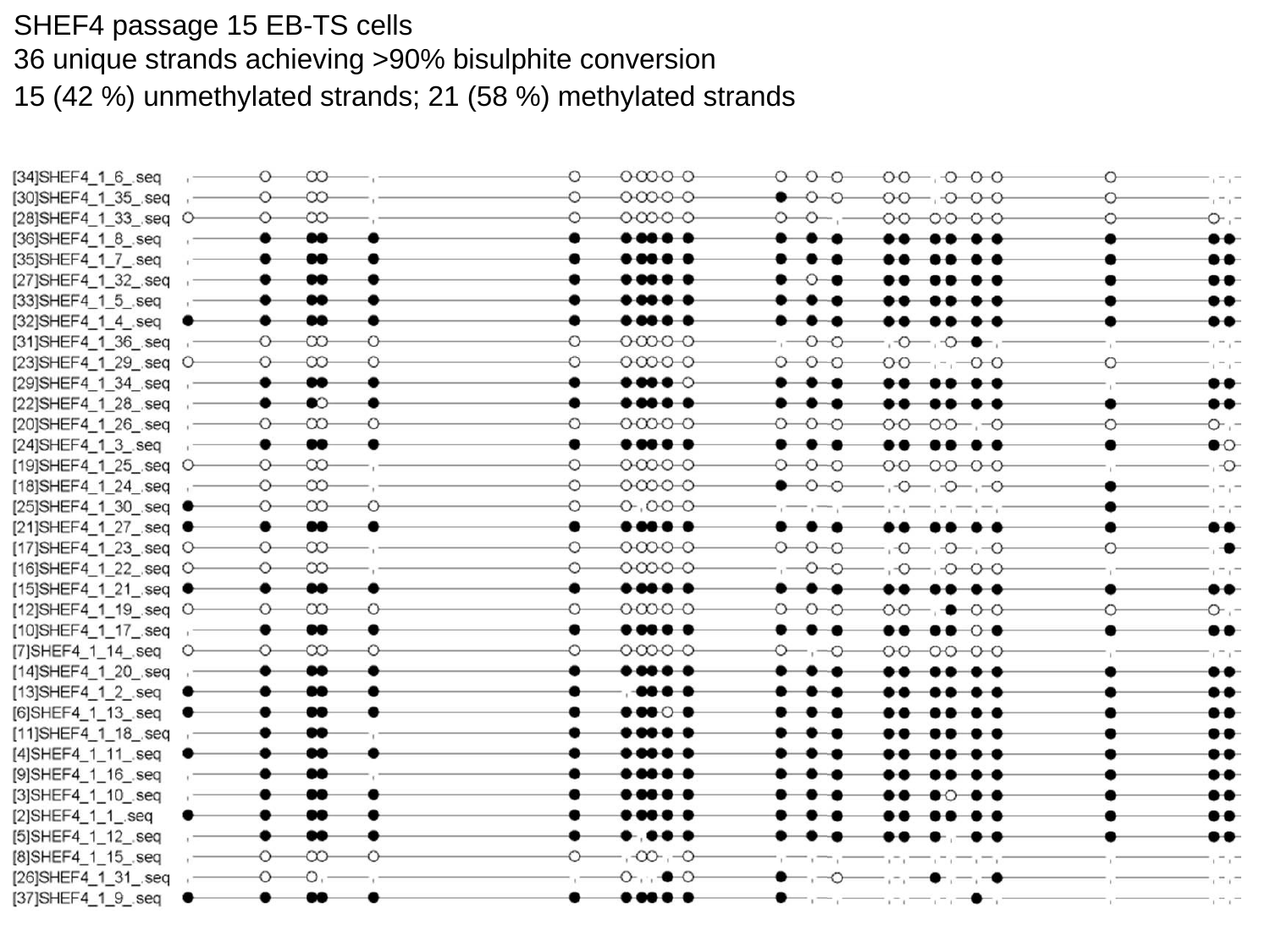

SHEF4 passage 15 EB-TS cells
36 unique strands achieving >90% bisulphite conversion
15 (42 %) unmethylated strands; 21 (58 %) methylated strands

## Slide 12
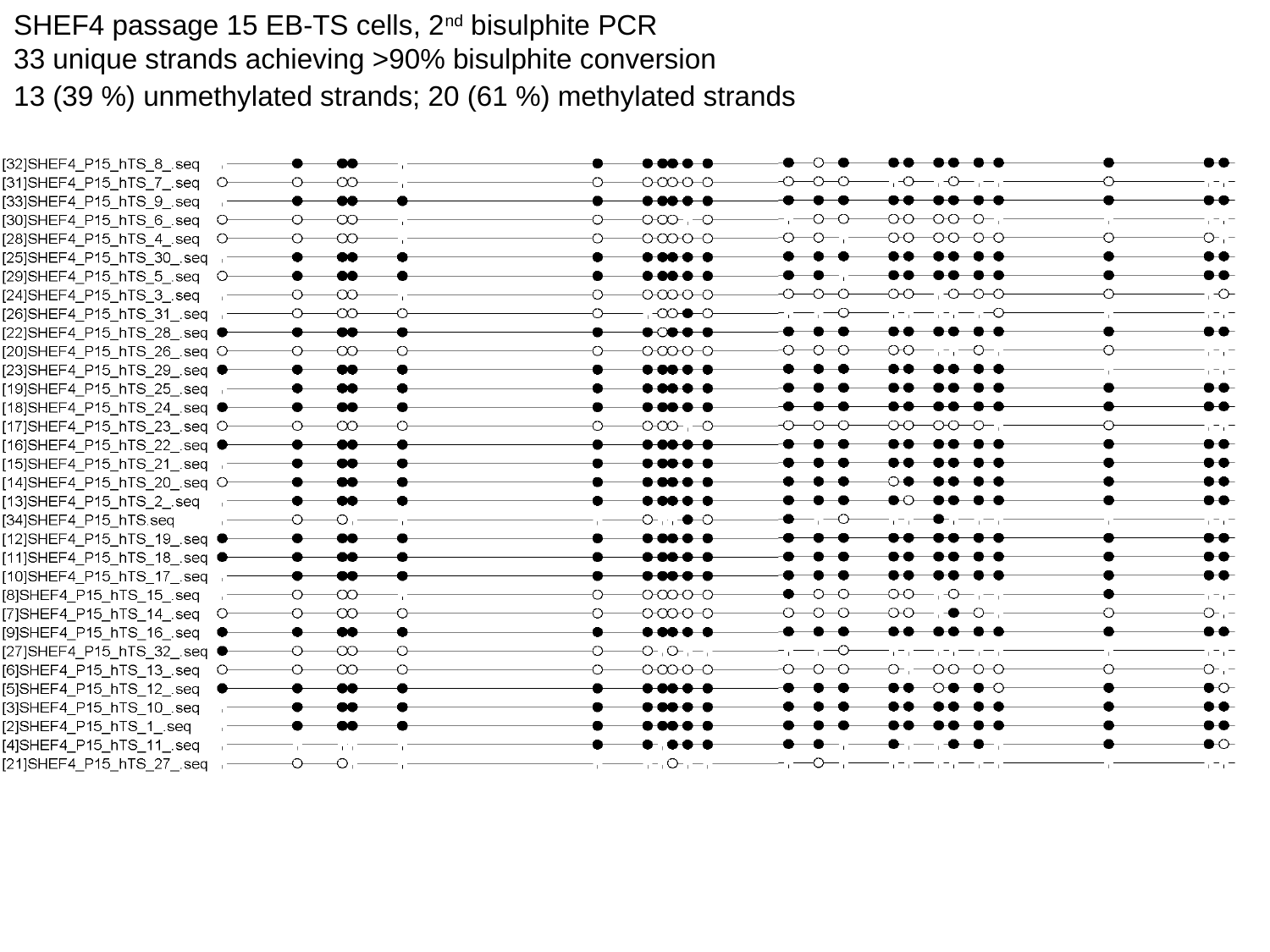

SHEF4 passage 15 EB-TS cells, 2nd bisulphite PCR
33 unique strands achieving >90% bisulphite conversion
13 (39 %) unmethylated strands; 20 (61 %) methylated strands
